# Draft Genome Sequence of Medusavirus Stheno, Isolated from the Tatakai River of Uji, Japan

**DOI:** 10.1128/MRA.01323-20

**Published:** 2021-01-07

**Authors:** Koki Yoshida, Ruixuan Zhang, Kimberly G. Garcia, Hisashi Endo, Yasuhiro Gotoh, Tetsuya Hayashi, Masaharu Takemura, Hiroyuki Ogata

**Affiliations:** a Bioinformatics Center, Institute for Chemical Research, Kyoto University, Gokasho, Uji, Japan; b Department of Bacteriology, Faculty of Medical Sciences, Kyushu University, Higashi-ku, Fukuoka, Japan; c Laboratory of Biology, Department of Liberal Arts, Faculty of Science, Tokyo University of Science, Shinjuku, Tokyo, Japan; KU Leuven

## Abstract

“*Medusaviridae*” is a proposed family of large double-stranded DNA (dsDNA) viruses so far represented by a sole virus isolated from a hot spring. In the present study, we report the isolation and genome sequencing of a second member of this family, medusavirus stheno, discovered from a freshwater sample with an Acanthamoeba castellanii coculture.

## Abstract (precis)

“*Medusaviridae*” is a proposed family of large double-stranded DNA (dsDNA) viruses so far represented by a sole virus isolated from a hot spring. In the present study, we report the isolation and genome sequencing of a second member of this family, Medusavirus stheno, discovered from a freshwater sample with an Acanthamoeba castellanii coculture.

## ANNOUNCEMENT

Medusavirus, the founding member of “*Medusaviridae*” of the phylum *Nucleocytoviricota*, was previously isolated from a hot spring in Japan (1). We isolated its relative named medusavirus stheno from freshwater sediment samples from the Tatakai River in Uji, Japan.

**FIG 1:**
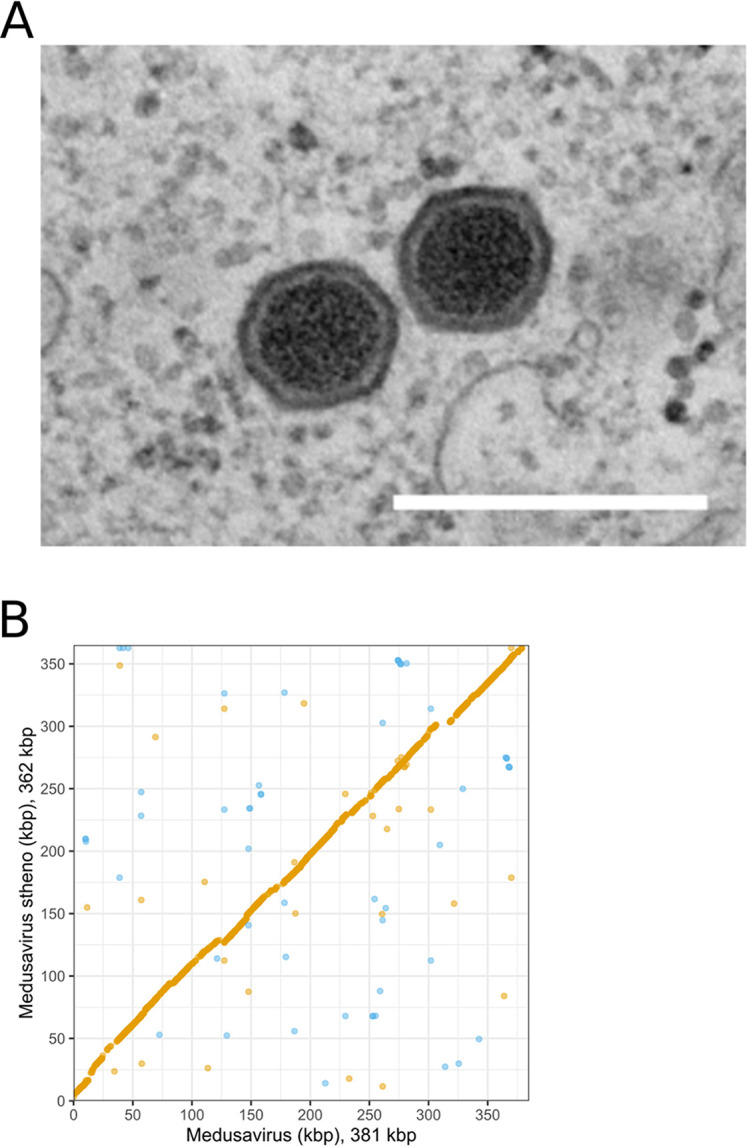
Medusavirus stheno virions and genome comparison. (A) Observation of medusavirus stheno virions by transmission electron microscopy. The experimental procedure was previously described in references 9 and 10. Bar = 400 nm. (B) Genome comparison between medusavirus and medusavirus stheno. The dot plot was generated using MUMmer v3.23 (11). Forward matches are colored orange, and reverse matches are colored blue. The long stretches of orange lines in the diagonal indicate a high conservation of gene order between medusavirus and medusavirus stheno.

Samples were filtered with filter paper 43 (Whatman PLC) and a 1.2-µm-pore-size Minisart syringe filter (Sartorius). Then, 90 µl of solution (18 ml of peptone yeast extract-glucose [PYG], 500 µl of amoeba cells [∼150 cells/µl]) and 9.5 µl of filtered samples were added in a 96-well plate. After 7 days of culture (26°C), 10 µl of supernatant from each well showing delayed proliferation under microscopic observation was mixed with 1 ml of PYG and 3 drops of amoeba culture solution in a 24-well plate. After 7 days, the supernatant from wells showing delayed proliferation was serially diluted down to 10^−11^-fold with PYG. Then, 10 µl of each diluted solution was mixed with 90 µl of PYG medium (16 ml PYG and 300 µl amoeba cells) in a 96-well plate. After 7 days, fresh amoeba cells were inoculated with the supernatants from wells showing delayed growth with at least 10^−6^-fold dilution in a 75-cm^2^ culture flask. After 2 days, culture solutions were centrifuged twice at 538 × *g* for 5 min at 26°C, and their supernatant was centrifuged at 8,000 × *g* for 35 min at 4°C. The resulting pellets were resuspended with 1 ml of phosphate-buffered saline. Centrifugation and resuspension were repeated twice to obtain pellets containing viral particles. Figure 1A shows virions in amoeba cells.

We extracted DNA from the pellets with a NucleoSpin tissue XS kit (Macherey-Nagel GmbH and Co. KG) and used Nanopore MinION (Oxford Nanopore Technologies, Inc.; ligation sequencing kit; 22,684 reads; minimum, 197 bp; median, 1,466.5 bp; maximum, 80,460 bp) and MiSeq (Illumina, Inc.; QIAseq FX DNA library kit; 120,066 reads; 2 × 151 bp) instruments for sequencing. Quality control of reads was performed with Trimmomatic v0.38 (5-base-wide window; quality threshold, ≥20; min_base_length, ≥35 bp) (2). Unicycler (SPAdes assembler, Pilon polisher) and Bandage were used for hybrid assembly (3, 4), producing a 362,811-bp contig (G+C content, 62.64%). Coding sequences (CDSs) were predicted using Prodigal (5) and GeneWise v2.4.1 (6). Promoter motifs in the 150-bp upstream regions of CDSs were identified using MEME v5.1.1 (E value, <10^−50^) (7). Sequence similarity searches (E value, <10^−5^) were performed against the NCBI nonredundant (NCBI NR) database using BLASTP (8).

The contig was colinearly aligned with the medusavirus genome (381 kb, average nucleotide identity of 79.8%; Fig. 1B) and found to encode 429 CDSs, 349 of which (81%) had their best hit in medusavirus and 21 of which had best hits in other viruses, amoebae, or other organisms. Like medusavirus, medusavirus stheno carried a complete set of histone domains (H1, H2A, H2B, H3, and H4); however, H3 and H4 fused into a single CDS. Two conserved sequence motifs were identified in the upstream regions of the CDSs (Table 1). These motifs were also found in the medusavirus genome.

**TABLE 1:** Sequence motifs identified in the upstream regions of medusaviruses

Virus and consensus sequence	No. of sites	E value
Medusavirus stheno		
SRCCAYATGAMBTCACATGGC	43	4.1e-162
VMMMAMADMAAMAAA	252	5.5e-130
		
Medusavirus		
GCCATRTGAVKTCATRTGGYSRSG	53	8.4e-183
VMAAMAAMARMAAMA	251	3.1e-146

### Data availability.

The sequence data are in DDBJ (DRA010707) and GenBank (MW018138).
